# Characterization of newly diagnosed type 1 diabetes in children and adolescents from 2017 to 2022 in China: a single-center analysis

**DOI:** 10.1186/s12887-023-04498-w

**Published:** 2024-01-04

**Authors:** Shimin Wu, Yuan Gao, Shusen Guo, Lina Fu, Yanqin Ying, Wei Wu, Ling Hou, Yan Liang, Xiaoping Luo

**Affiliations:** Department of Pediatrics, Tongji Hospital, Tongji Medical College, Huazhong University of Science and Technology, Wuhan, China

**Keywords:** Newly diagnosed type 1 diabetes, Autoimmunity, Ketoacidosis, Children and adolescents, COVID-19

## Abstract

**Objective:**

This study investigated the characteristics of newly diagnosed type 1 diabetes mellitus (T1DM) related to autoimmunity and the frequency of diabetic ketoacidosis (DKA) in children and adolescents from 2017–2022 in China.

**Research design and methods:**

Single-center regional data from the Department of Pediatric Endocrinology, Tongji Hospital, were used to compare 88 children and adolescents newly diagnosed with T1DM from 2020 to 2022 (i.e. during the COVID-19 pandemic in China) and 76 children and adolescents diagnosed with T1DM from 2017 to 2019. Auto-antibodies, including glutamic acid decarboxylase-65 and insulin auto-antibodies, were detected by enzyme-linked immunoassays. DKA was defined as a pH < 7.3 and/or a bicarbonate level < 15 mmol/L.

**Results:**

The median age of the 164 children and adolescents newly diagnosed with T1DM from 2017 to 2022 was 7.0 years (interquartile range [IQR]: 3.8–10.0 years; 51.83% male). The mean annual incidence of T1DM was 2.98 per 1,000,000 child years. The estimated frequency of auto-antibody positivity was 51.22% (*n* = 84), and there was no difference between the 2020–2022 group and 2017–2019 group (55.68% [*n* = 49] vs. 46.5% [*n* = 35]; *p* = 0.219). The frequency of DKA among the entire cohort was 57.93% (*n* = 95), and peaked in 2020 at 78.9% (15/19 patients). The frequency of DKA was not significantly higher in the 2020–2022 group compared with the 2017–2019 group (60.23% [*n* = 53] vs. 55.26% [*n* = 42]; *p* = 0.521). We found no significant difference in the frequency of DKA between patients who were negative vs. positive for auto-antibodies in the 2020–2022 group (64.10% [*n* = 25] vs. 57.14% [*n* = 28], *p* > 0.05). The C-peptide level and HbA1c (%) were positively correlated with onset age (R1 = 0.389, *p* < 0.01; R2 = 0.371, *p* < 0.01), and the estimated mean C-peptide level was 0.26 ng/ml (IQR: 0.2–0.4 ng/ml) in patients with DKA and 0.370 ng/ml (IQR: 0.2–0.6 ng/ml) in patients without DKA (*p* = 0.044).

**Conclusions:**

This study showed the annual incidence of T1DM was 2.98 per 1,000,000 child years, gradually increased over the study period, and there was no significant increase in T1DM with auto-antibody positivity in children and adolescents newly diagnosed from 2020–2022 in China compared with the previous 3 years. Furthermore, the frequency of DKA was peaked in 2020, and were not significantly different between patients who were negative vs. positive for auto-antibodies.

## Background

Type 1 diabetes mellitus (T1DM) is the most common type of diabetes in children and adolescents [1]. In most cases, it is caused by the autoimmune destruction of pancreatic β-cells resulting in insulin deficiency and hyperglycaemia [2]. T1DM is subdivided into two immunophenotypic categories, auto-antibody positive and auto-antibody negative, based on the presence of detectable serum auto-antibodies [3]. Most T1DM patients are positive for one or more islet cell auto-antibodies, including glutamic acid decarboxylase-65 antibodies (GADA-65), insulinoma antigen-2 antibodies (IA-2A), insulin auto-antibodies (IAA), Zinc transporter 8 antibodies (ZnT8A), and islet cell antibodies (ICA) [3]. The incidence of type 1 diabetes in children and adolescents has been increasing worldwide, and roughly 80,000 children < 15 years of age develop T1DM each year [2]. Diabetic ketoacidosis (DKA) is the most serious complication of T1DM in children and adolescents and it carries a significant risk of morbidity and/or mortality [4].

Infection with severe acute respiratory syndrome coronavirus 2 (SARS-CoV-2), the causative agent of the pandemic coronavirus disease 2019 (COVID-19), was posited to lead to the development of diabetes through direct cytotoxicity to β-cells, without a role for autoimmunity [5, 6]. Previous studies identified positive immunostaining in pancreatic islets for ACE2, the main receptor for SARS-CoV-2 [7, 8]. This led to the hypothesis that SARS-CoV-1 tropism for β-cells could directly damage pancreatic islets [7, 8]. Several studies reported a significant increase in newly diagnosed T1DM and a greater proportion of patients presenting with DKA at diagnosis during the COVID-19 pandemic compared to prior years, while others found no change in the expected or actual numbers of newly diagnosed T1DM patients [9–12]. Thus, whether the rate of new T1DM diagnoses and the proportion of patients with DKA at diagnosis increased during the COVID-19 pandemic is still controversial.

Relatively few children and adolescents in China were infected with COVID-19, and data on the numbers of children and adolescents with newly diagnosed T1DM from 2020–2022 are limited. Thus, the aim of this study was to investigate the annual incidence of T1DM and the characteristics of newly diagnosed T1DM with respect to autoimmunity and the frequencies of DKA in children and adolescents from 2017–2022 in China.

## Methods

### Data source and study population

This study was a retrospective chart review of all paediatric (≤ 14 years old) patients with newly diagnosed T1DM. Electronic medical record data were collected from patients seen at the Department of Pediatric Endocrinology, Tongji Hospital, from January 2017 to December 2022. The inclusion criteria for T1DM followed the standards of medical care for diabetes of the American Diabetes Association [2, 13]. The diagnosis of DKA was based on recent International Society for Pediatric and Adolescent Diabetes guidelines: hyperglycaemia (blood glucose ≥ 11 mmol/L), metabolic acidosis (venous blood pH < 7.3 or serum bicarbonate < 15 mmol/L), and ketosis (presence of ketones in the blood or urine) [4, 14]. According to the WHO reference intervals, age- and gender- independent height and weight z-scores were calculated as previously described [15, 16]. Body mass index (BMI) was calculated as weight (kg)/height (m^2^), and was converted to a BMI Z-score according to the LMS method: Z = [(BMI/M)L − 1]/(L × S) [the median (M), coefficient of variation (S) and skewness (L)] [17]. Obesity was defined as ≥ 95th percentile of gender-specific BMI according to the growth charts of children in China [18]. The incidence of T1DM was calculated by summing the numbers of children and adolescents newly diagnosed in Tongji hospital each year, and then dividing by the total general population (≤ 14 years old) in Hubei Province, China (the data are mainly collected from Hubei Statistical Yearbook, http://tjj.hubei.gov.cn/).

### Variables

Demographic data included age at diabetes onset and sex. Clinical data included random blood glucose, haemoglobin A1c (HbA1c), pH, fasting C-peptide, and islet auto-antibodies (GADA-65 and IAA), measured at diagnosis at our hospital. HbA1c levels were determined in the clinical laboratory using high-performance liquid chromatography (Bio-Rad, Hercules, CA, USA). C-peptide and islet auto-antibodies were measured by the endocrinology laboratory (Tongji Hospital, Wuhan, China). C-peptide was measured using a solid-phase, two-site chemiluminescence immunometric assay. Islet auto-antibodies were detected using a radioligand assay according to the islet auto-antibody standard procedure. The presence of COVID-19 was detected using RT-PCR in the clinical laboratory.

### Statistical analysis

Data were analysed using IBM SPSS software version 22 (IBM Corp., Armonk, NY, USA). Outcomes are presented as the median and inter-quartile range (IQR) for the description of continuous variables and as a percentage for the description of categorical variables. Comparisons between groups were performed using t-tests, the Wilcoxon rank sum test for continuous outcomes, and the χ2 test for dichotomous outcomes. Pearson’s test was used for correlation analysis. The level of significance was set at *p* < 0.05.

## Results

**Fig. 1:**
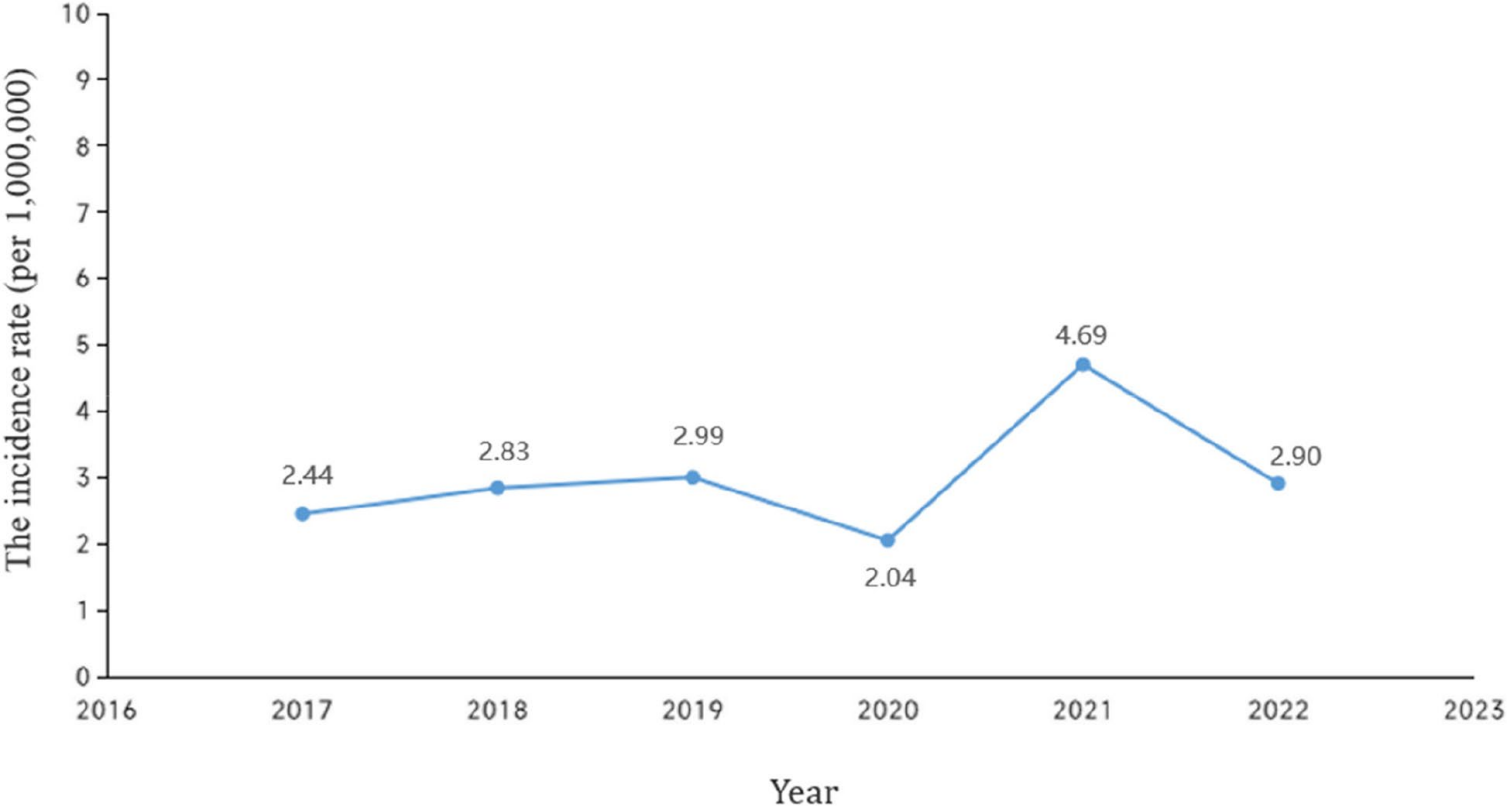
The incidence of T1DM in children and adolescents from 2017–2022

The study population consisted of 164 paediatric patients (85 males and 79 females) newly diagnosed with T1DM between January 2017 and December 2022. The annual incidence of T1DM in children and adolescents aged 0–14 years gradually increased over the study period (Fig. 1). It was 4.69/100,000 (43/9,170,000) at its peak in 2021, while it was 2.90/1,000,000 (26/8,960,000) declining in 2022. The mean annual incidence of T1DM was 2.98 per 1,000,000 child years, and the compound annual growth rate (CAGR) was 2.92% during the period 2017–2022. The median age of the cohort was 7.0 years (IQR: 3.8–10.0 years). There were no significant differences between the 2017–2019 and 2020–2022 groups with respect to sex, age at diabetes onset, serum C-peptide levels, and %HbA1c (Table 1). Moreover, the 2020–2022 group was not infected with COVID-19 at the onset of T1DM, and these patients or their parents had stated that they had not been infected with COVID-19 during the pandemic.

**Table 1:** Characteristics of patients with newly diagnosed T1D in the 2020–2022 cohort versus the 2017–2019 cohort

Characteristics	Group A (2020–2022 year)	Group B (2017–2019 year)	*p*-Value
Newly diagnosed T1D patients	88	76	-
Age of onset (years), median (IQR)	7.0 (3.5,10.0)	6.050 (3.8,10.0)	0.842
Male sex, n (%)	45 (51.14)	40 (52.63)	0.848
Height (cm)	124.60 ± 25.41	124.88 ± 19.16	0.956
Height Z-score	0.49 ± 1.26	0.16 ± 1.47	0.261
Weight (kg)	22.00 (15.0,29.5)	21.00 (16.0,30.8)	0.915
Weight Z-score	-0.030(-0.9,0.8)	-0.205(-0.9,0.5)	0.439
BMI (kg/m^2^)	15.86 ± 1.99	15.29 ± 1.70	0.173
BMI Z-score	-0.25 ± 1.23	-0.43 ± 1.06	0.483
COVID-19 Nucleic acid detection	Negative	-	-
Random glucose level (mmol/L)	28.80 (22.1,32.1)	28.350 (21.9,33.3)	0.456
Fasting insulin level (uIU/ml)	1.60 (1.2,2.8)	1.60 (1.2,2.1)	0.587
Fasting C-peptide (ng/ml)	0.28 (0.2,0.4)	0.34 (0.2,0.6)	0.156
HbA1c (%)	12.57 ± 2.35	12.43 ± 2.14	0.691
DKA on presentation n (%)	53 (60.23)	42 (55.26)	0.521
Diabetes-Associated Auto-antibodies (one or more antibody positive), n (%)	49 (55.68)	35 (46.05)	0.219
Anti-GAD65 n (%)	43 (48.86)	26 (34.21)	0.058
IAA n (%)	19 (21.59)	15 (19.74)	0.770
Treatments (Intensive insulin therapy/Insulin pump therapy)	54/34	48/28	-

Auto-antibody measurements were available for all 164 patients (100%). In 51.22% (*n* = 84), positivity for one or more serum antibodies was detected, including GADA (42.07%, *n* = 69) and IAA (20.73%, *n* = 34) (Table 1). A comparison of patients with positive and negative auto-antibody test results from both periods showed estimated frequencies of auto-antibody positivity of 55.68% (*n* = 49) and 46.05% (*n* = 35) in 2020–2022 and 2017–2019, respectively (*p* > 0.05).

**Fig. 2:**
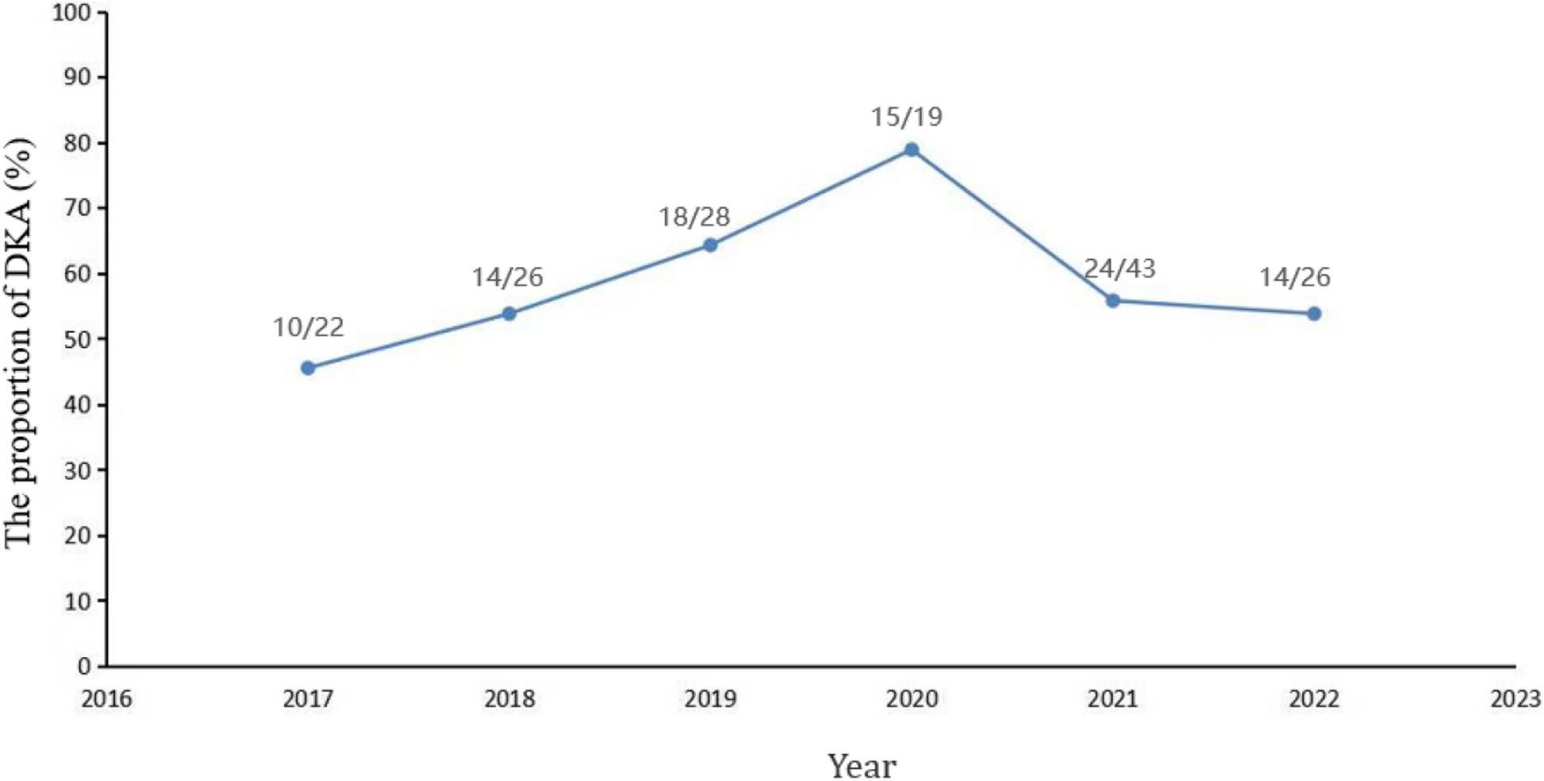
The proportion of DKA in patients with new onset T1DM from 2017–2022

**Fig. 3:**
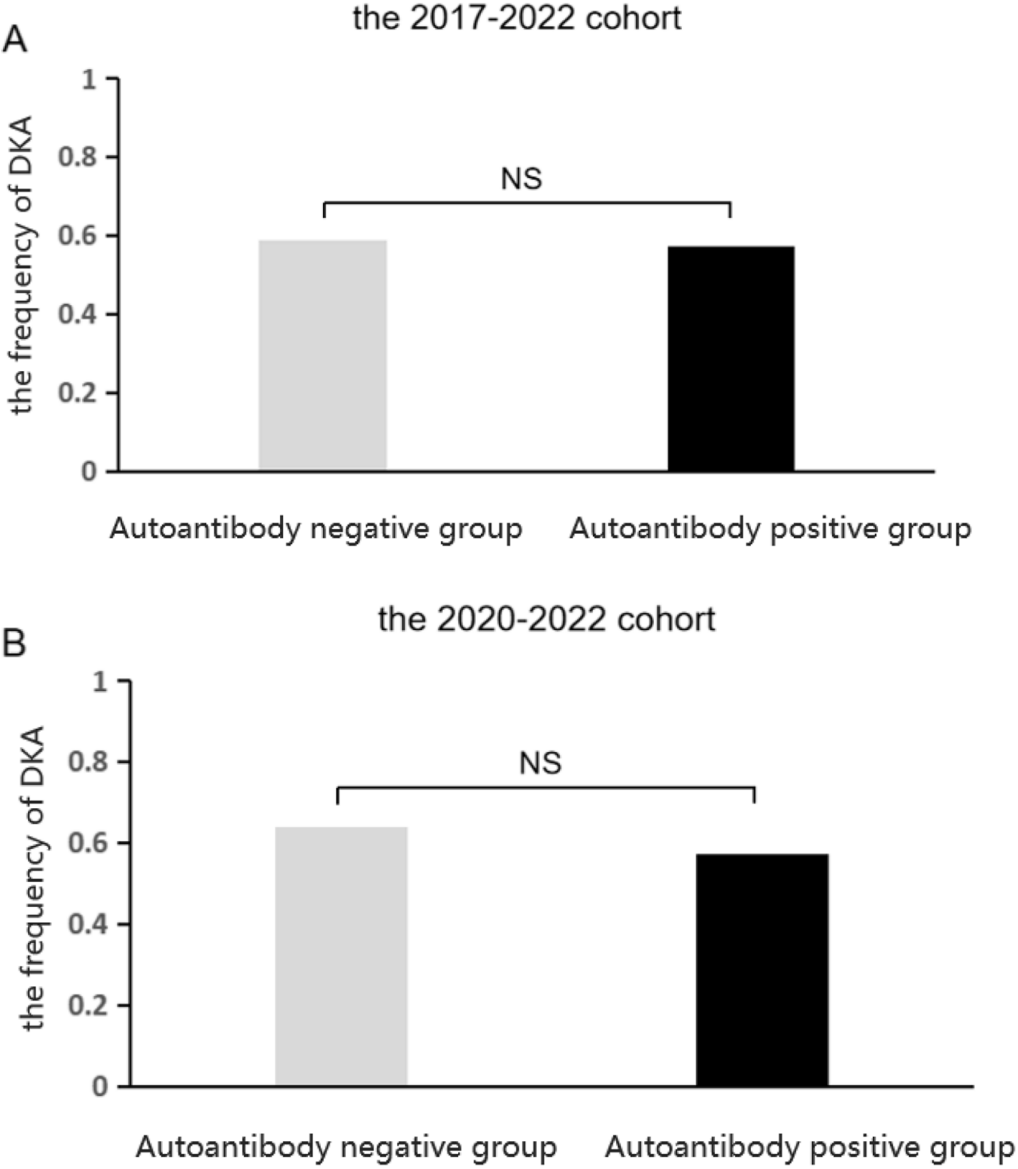
The frequencies of DKA between patients who were negative vs. positive for auto-antibodies in the 2017–2022 cohort (58.75% [*n* = 47] vs. 57.14% [*n* = 48], NS *p* > 0.05) and the 2020–2022 cohort (64.10% [*n* = 25] vs. 57.14% [*n* = 28], NS *p* > 0.05)

The proportion with DKA among all 164 patients was 57.93%. The proportion of patients who presented in DKA over the year 2017–2022 fluctuated from year to year, and peaked in 2020, the first year of COVID, at 78.9% (15/19 patients) (Fig. 2). Moreover, the frequency of DKA was not significantly higher in the 2020–2022 group compared with the 2017–2019 group (60.23% [*n* = 53] vs. 55.26% [*n* = 42]; *p* = 0.521, Table 1). And the frequency was not significantly different between patients who were negative vs. positive for auto-antibodies in the 2017–2022 cohort (58.75% [*n* = 47] vs. 57.14% [*n* = 48], *p* > 0.05, Fig. 3A) or 2020–2022 cohort (64.10% [*n* = 25] vs. 57.14% [*n* = 28], *p* > 0.05, Fig. 3B).

**Fig. 4:**
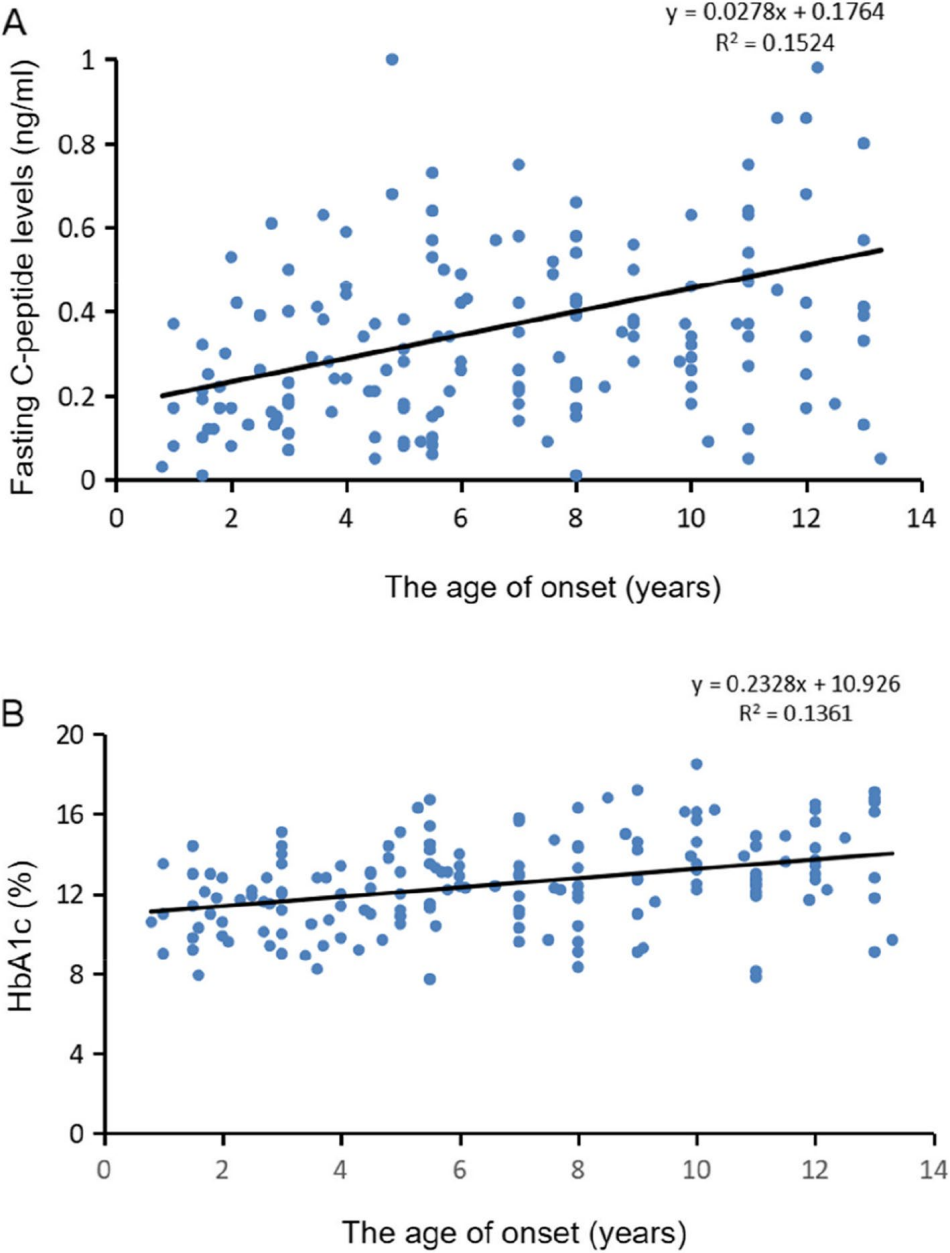
C-peptide level and HbA1c (%) were positively correlated with the age of onset in the all cohort (R1 = 0.389, *p* < 0.01 and R2 = 0.371, *p* < 0.01)

**Fig. 5:**
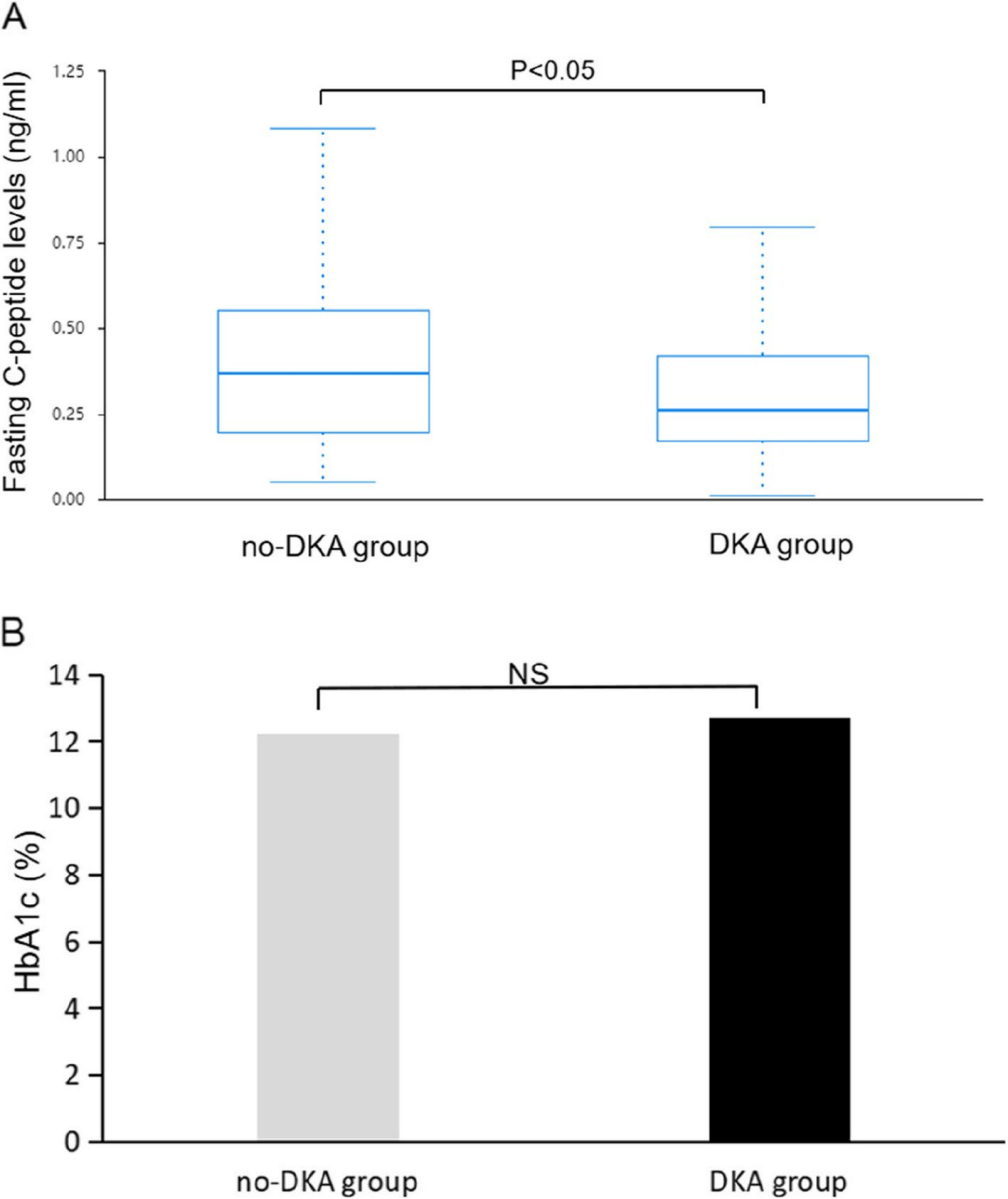
C-peptide level (**A**) and HbA1c (%) (**B**) in no-DKA group and DKA group from 2017–2022. A the C-peptide level in patients with DKA was lower than it in patients without DKA (0.26 ng/ml vs. 0.370 ng/ml, *p* = 0.044). B HbA1c (%) was no difference in no-DKA group and DKA group ( 12.21 ± 2.16 vs.12.72 ± 2.29. *p* > 0.05)

As shown in Fig. 4, in all patients, the fasting C-peptide level and HbA1c (%) were positively correlated with the onset age (R1 = 0.389, *p* < 0.01 and R2 = 0.371, *p* < 0.01). The estimated mean C-peptide level was 0.26 ng/ml (IQR: 0.2–0.4 ng/ml) in patients with DKA and 0.370 ng/ml (IQR: 0.2–0.6 ng/ml) in patients without DKA (*p* = 0.044), and HbA1c (%) were no differences in no-DKA group and DKA group (*p* > 0.05, Fig. 5). In addition, the estimated mean C-peptide level and HbA1c (%) were not differences in the auto-antibody positivity and negativity groups from 2017–2022 (*p* > 0.05).

## Discussion

We found that the mean annual incidence of T1DM in children and adolescents in Hubei Province in 2017–2022 was 2.98 per 1,000,000 child years, which was mildly lower than the average incidence of T1DM in China (0.1 ~ 3.03/100,0000) [19–21]. The low incidence in Hubei is probably attributed to genetic, environmental, regional, and behavioural factors [19]. In addition, this rate was gradually increased by 2.92% over the study period, and it is consistent with previous studies conducted in the world [22]. However, in 2021, the incidence of T1DM in our data was markedly elevated, while it was decreased in 2022. Additionally, the number of patients with newly diagnosed T1DM who were not infected with COVID-19 increased significantly in 2021, although the reason was unclear. A recent longitudinal study involving multiple institutions across the United States reported a significant increase in the risk of both type 1 and type 2 diabetes after COVID-19 infection; there were 1,399 newly diagnosed T1DM patients in 2020 and 1,277 in 2019 [23, 24]. This mirrors the increased incidence of T1DM following a Coxsackievirus B5 epidemic in the 1980s [25]. Future longitudinal studies with large population-level datasets are needed to determine the long-term impact of COVID-19 on diabetes trends.

Although the potential viral aetiology of T1DM has been extensively explored, information regarding causality is still lacking [26]. During the COVID-19 pandemic, an increase in newly diagnosed T1DM was predicted due to the virus’s affinity for the ACE2 protein rather than to the development of auto-antibodies [6, 7]. However, studies examining the first wave of the pandemic found no evidence of a significant increase in the number of new cases of auto-antibody-negative T1DM in children, adolescents, and young adults, although the sample sizes were small and the data were limited to patients in the UK, Germany, and Italy [9, 11]. Viral infections play an important role in the pathogenesis of T1DM and can determine if a genetically susceptible individual develops the disease [25]. Indeed, viral infection itself may accelerate the clinical course in patients previously established to be at risk (Ab-positive) [26]. In our study, the number of newly diagnosed T1DM patients with auto-antibody positivity did not significantly increase in children and adolescents during the COVID-19 pandemic in China compared to the years preceding the pandemic. Moreover, during the COVID-19 pandemic there was no significant increase in newly diagnosed T1DM with auto-antibody negativity, although this may have been related to the small number of infected children and adolescents (~ 171 patients) and the low incidence of T1DM in children and adolescents in China [19–21, 27]. It is not clear whether COVID-19 infection triggers late adaptive immunity, which results in progressive pancreatic β cell death and an accelerated clinical course in patients with positive antibodies. Further research is necessary to address this possibility.

Insulin deficiency can cause DKA, a serious complication of T1DM characterized by the triad of hyperglycaemia, acidosis, and ketosis [4]. In some countries, studies have shown an increase in the proportion of newly diagnosed T1DM patients presenting with serious DKA during the COVID-19 pandemic compared to previous years [11, 12, 23, 28–30]. This can be explained by delays in seeking medical care, especially during the early months of the pandemic [15, 25]. However, the frequency of DKA was not higher in patients with idiopathic T1DM than in patients with immune-mediated T1DM, contrary to the hypothesis that SARS-CoV-2 infection leads to a significant increase in newly diagnosed autoimmune-negative T1DM [11]. In this study, we found a significant increase in the frequency of DKA at the first year of the COVID-19 epidemic, and then decreased graduall during the COVID-19 pandemic, while the trend was consistent with the frequencies of DKA at the onset of T1DM in other countries [11, 12, 23, 28–30]. Consistent with the above-mentioned studies and with the DKA rates reported in the literature, in our patients there was no significant increase in the frequency of DKA in patients with idiopathic vs. immune-mediated T1DM [11]. In addition, we found that the estimated mean C-peptide level was lower in patients with versus without DKA (*p* = 0.044) in the entire cohort, and that fasting C-peptide levels and HbA1c (%) were positively correlated with the onset age. Thus, the C-peptide level may serve as a marker of the occurrence of DKA in children and adolescents with type 1 diabetes.

The limitations to this study include its retrospective design and the lack of data from a national multi-center study. Furthermore, our sample size was not large enough to determine the incidence of T1DM in the general population between the pre-pandemic and post-pandemic years.

## Conclusions

In summary, this study showed the annual incidence of T1DM was 2.98/1,000,000, gradually increased over the study period, and there was no significant increase in newly diagnosed T1DM with auto-antibody positivity in children and adolescents from 2020–2022 in China compared with previous years. Moreover, we found the frequency of DKA was peaked in 2020, and there was no significant difference in the frequency of DKA in children or adolescents who were negative versus positive for auto-antibodies in the entire study cohort.

## Data Availability

The datasets used in this study are available from the corresponding author on reasonable request.
